# Recent advances in understanding the regulatory mechanisms of P4-ATPase lipid flippases

**DOI:** 10.1042/BST20260203

**Published:** 2026-10-05

**Authors:** Pauline Funke, Mathilde Roth, Thibaud Dieudonné, Guillaume Lenoir

**Affiliations:** Université Paris-Saclay, CEA, CNRS, Institute for Integrative Biology of the Cell (I2BC), Gif-sur-Yvette, France

**Keywords:** cryo-electron microscopy, lipids, phosphatidylinositol, phosphorylation/dephosphorylation, protein-protein interactions, transport

## Abstract

P4-ATPases establish and maintain transbilayer lipid asymmetry in eukaryotic membranes by transporting phospholipids from the exoplasmic to the cytosolic membrane leaflet at the expense of ATP hydrolysis. In recent years, biochemical analysis together with an increasing number of cryo-electron microscopy structures has substantially increased our understanding of the lipid transport cycle and substrate recognition by P4-ATPases. However, the underlying molecular mechanism governing P4-ATPase activation and regulation remains less well understood. The present review aims to summarize current insights into the molecular mechanisms controlling P4-ATPase activation and regulation. We discuss autoinhibition by N- and C-terminal extensions, activation by regulatory lipids, particularly phosphoinositides, regulation by interacting proteins including small GTPases and guanine nucleotide exchange factors, and post-translational modifications, especially phosphorylation. Together, these studies reveal that P4-ATPase activity is controlled by a complex and multilayered regulatory network that enables precise control of lipid transport. Future studies combining structural, biochemical, and cellular approaches will be essential to elucidate how these mechanisms cooperate to maintain membrane lipid homeostasis and support diverse cellular functions.

## Introduction

Eukaryotic membranes display an asymmetric lipid distribution, with negatively charged lipids such as phosphatidylserine (PS) and phosphatidylinositol (PI) primarily confined to the cytosolic leaflet (e.g., [1–3].). The maintenance of transbilayer lipid asymmetry, as well as its controlled disruption, is crucial for many cellular processes, such as protein recruitment to the membrane or in apoptosis [4,5].

Lipid flippases, which mostly belong to the P4 subfamily of P-type ATPases (P4-ATPases), catalyze lipid transport from the exoplasmic to the cytosolic membrane leaflet at the expense of ATP hydrolysis, thereby actively contributing to the establishment and maintenance of lipid asymmetry [6]. P4-ATPases are indispensable for normal cellular function, and mutations in mammalian P4-ATPases have been implicated in numerous pathological conditions, including blood, hepatic, and neurological disorders [7].

Advances in cryo-electron microscopy (cryoEM) have heralded tremendous progress in the structural characterization of P4-ATPases in various states of their catalytic cycle [8–14]. P4-ATPases are composed of ten transmembrane (TM) helices and three catalytic domains located in the cytosol: the nucleotide binding (N) domain that binds ATP, the phosphorylation (P) domain containing a conserved aspartate residue that becomes phosphorylated during the transport cycle, and the actuator (A) domain that catalyzes dephosphorylation of the P-domain [9] (Figure 1A). Notably, P4-ATPases from the A and C clades form a heterodimeric complex with a CDC50 protein that is required for their subcellular localization and catalytic function [15–17] (Figure 1A). In contrast, P4B-ATPases function as monomers [11,18,19].

**Figure 1:**
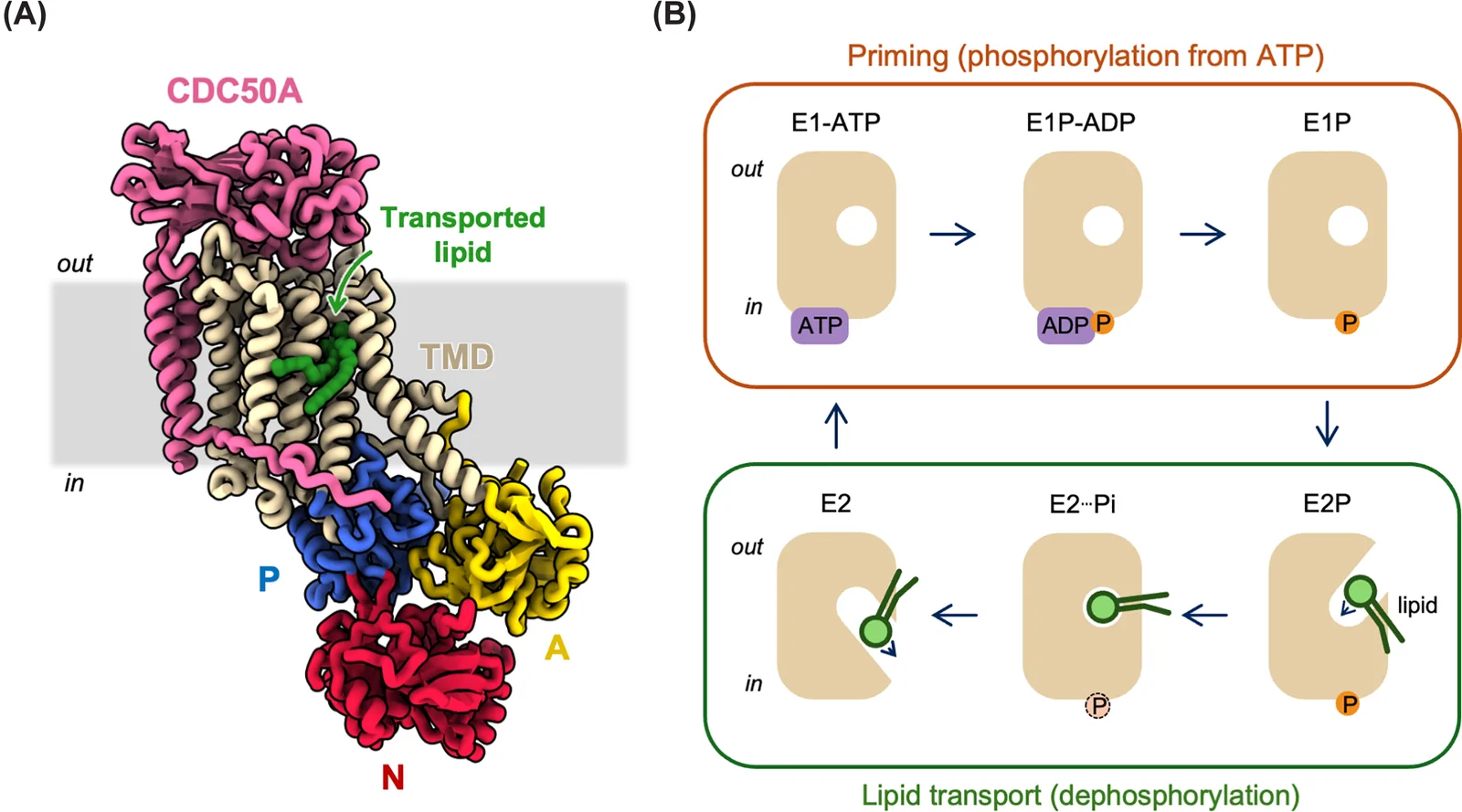
Structure and lipid transport cycle of P4-ATPases (**A**) Structure of the human ATP8B1-CDC50A flippase complex captured during lipid translocation (E2^…^Pi conformation). The cytosolic A-, N-, and P-domains of ATP8B1 are colored yellow, red, and blue, respectively. The transmembrane domain (TMD) of ATP8B1 is shown in wheat. The transported lipid, occluded within the TMD of ATP8B1, is shown in green. The CDC50A subunit is colored pink. (PDB: 8OXC [14]). (**B**) P4-ATPase lipid transport cycle. The catalytic cycle of P4-ATPases can be divided into two main phases. The first, referred to as the priming phase, involves ATP-dependent phosphorylation of the ATPase, generating the high-energy E1P state. This step is independent of any lipid binding at the transport site. The second corresponds to the lipid transport phase, during which relaxation of the high-energy state first leads to the outward-open E2P conformation, competent for lipid binding. Lipid recognition and occlusion in the E2^…^Pi state then trigger enzyme dephosphorylation, resulting in the release of the lipid headgroup on the cytosolic side.

P4-ATPase lipid transport is achieved through alternating phosphorylation and dephosphorylation events, as described by the Post–Albers reaction cycle [20,21] (Figure 1B). Binding of ATP to the E1 state drives phosphorylation of the catalytic aspartate in the highly conserved DKTGT motif of the P-domain, yielding the E1P state (P denotes phosphorylation). In this state, the outer gate, which the transported lipid initially enters before being translocated across the membrane, remains closed. Opening of the outer gate occurs during formation of the E2P intermediate and requires a large conformational change of the A-domain. Owing to its direct connection to the N-terminal region of the TMD, movement of the A-domain triggers the opening of the outer gate, allowing the phospholipid headgroup to access its binding site. Binding of the headgroup subsequently induces gate closure and substrate occlusion. This gate closure is transmitted back to the A-domain, where proper positioning of the glutamate's carboxyl group in the conserved DGES/T motif promotes water coordination for nucleophilic attack on the aspartyl-phosphate (E2^…^Pi transition state). The A-domain is then thought to disengage, resulting in the opening of the cytoplasmic gate in the E2 state [10,22]. Notably, the structure of this E2 intermediate with an open cytoplasmic gate has not yet been determined.

Although recent progress has been made in the structural characterization of P4-ATPases and the identification of cofactors required for lipid translocation, as well as in the biochemical characterization of their substrate specificity using cell- and proteoliposome-based assays, much less is known about the molecular mechanisms of how flippases are activated for lipid transport within the cellular environment and how this lipid transport is regulated. The present review aims to summarize our current understanding of the mechanisms governing P4-ATPase activation and regulation, with particular emphasis on autoinhibition mediated by the N- and C-terminal tails, the role of regulatory lipids and interacting proteins, and post-translational modifications—particularly phosphorylation.

## Autoinhibition by N- and/or C-terminal extensions of P4-ATPases

The cytosolic N- and C-terminal extensions of P4-ATPases are largely disordered, variable in their length, and with poor sequence conservation. Besides contributing to the subcellular localization of many P4-ATPases ([23], reviewed in [24]), the N- and C-terminal extensions can play an autoinhibitory role as well, thereby tightly regulating protein function. The best studied examples in this context are the yeast flippase complex Drs2-Cdc50 and its mammalian homologs ATP8B1-CDC50A and ATP8A2-CDC50A, all of which display markedly higher ATPase activity when truncated in their N- and/or C-terminal tail [25–28].

In the case of Drs2, Zhou and colleagues were the first to report that deletion of 121 C-terminal residues increased the ATPase activity of purified Drs2 about four-fold compared with the full-length protein [25]. Subsequent work on Drs2 further explored the role of its terminal tails [26]. ATPase activity of the purified protein was markedly stimulated upon removal of both the first 104 N-terminal residues and residues 1291–1355 at the C-terminus [26]. Subsequent analyses further narrowed the autoinhibitory region to C-terminal residues 1291–1302, as removal of only the last 53 residues of Drs2 (1302–1355) had little effect on ATPase activity [26]. Notably, C-terminal truncation had a more pronounced effect on Drs2 activation than removal of the 104 N-terminal residues, although full activation of the complex required truncation of both terminal extensions [26]. The contribution of the N- and C-terminal tails to Drs2 activation was further substantiated following reconstitution of the protein into proteoliposomes: only the doubly truncated version of Drs2, Drs2∆NC (residues 105–1247), was capable of catalyzing phospholipid transport across the liposomal membrane, not the full-length protein [29,30]. Similar observations were made for the ATP8B1-CDC50A complex. While the purified full-length protein can be activated to some extent in the presence of PS [31], ATPase activity is substantially higher upon removal of both N- and C-terminal residues (ATP8B1∆NC, residues 43–1174) [27]. The N-terminal extension cooperates with the C-terminal tail to mediate full autoinhibition of the ATP8B1-CDC50A complex. Consistent with this model, truncation of the ATP8B1 N-terminus has been shown to partially relieve autoinhibition, whereas truncation of its C-terminus results in a greater increase in enzymatic activity [31]. Using a trans-inhibition strategy with synthetic peptides derived from the C-terminal tail of ATP8B1, Laursen and co-workers further identified residues 1216–1228 as a key autoinhibitory region and demonstrated a specific contribution of the ionic interaction between R1228 and E219 in the A-domain. Interestingly, residues 1216–1228 of the ATP8B1 C-terminal tail overlap with residues 1291–1302 in Drs2, which bridges the A- and N-domains in the autoinhibited conformation [32].

What is the underlying molecular mechanism of autoinhibition? Structural studies of Drs2 and ATP8B1 provide important clues. In both proteins, the C-terminal tail adopts an extended conformation that runs along the catalytic N- and A-domains, establishing extensive interactions with both domains (Figure 2A). Notably, a conserved (G/A)(Y/F)AFS motif occupies the ATP binding site within the N-domain [9,27] (Figure 2B). This interaction has been proposed to sterically hinder ATP binding, thereby preventing the ‘priming’ of the protein via autophosphorylation of the E1 intermediate (Figure 1B) [13]. Such a model, however, conflicts with results from several other studies. First, full-length Drs2 is readily phosphorylated by ATP, indicating that the presence of the (G/A)(Y/F)AFS motif does not preclude ATP binding. Rather, the autoinhibitory C-terminal tail is likely to exist in a dynamic equilibrium between free and bound conformations, engaging only transiently with the cytosolic domains. Consistent with this view, the C-terminal tail has only been resolved in an inhibitory conformation in the E2P state [9,13,33], suggesting that this state displays the highest affinity for the autoinhibitory tail. Secondly, removing residues R1290–I1355 in Drs2, which encompass the entire region downstream of the (G/A)(Y/F)AFS motif, is sufficient to relieve autoinhibition (Figure 2B) [26]. Likewise, an ATP8B1 C-terminal peptide lacking the (G/A)(Y/F)AFS motif retains substantial inhibitory properties, although inclusion of the motif further strengthens inhibition [32]. Together, these observations suggest that the (G/A)(Y/F)AFS motif is not the main determinant of autoinhibition. Instead, autoinhibition primarily arises from interactions involving residues located downstream of the (G/A)(Y/F)AFS motif, particularly through contacts with the A-domain, whereas the motif itself acts mainly to reinforce the inhibitory state. This model would also be consistent with the notion that autoinhibition specifically impedes the E2P-to-E2 transition [26], which requires a characteristic repositioning of the A-domain upon substrate occlusion to promote efficient dephosphorylation of the catalytic aspartate (Figure 2C,D).

**Figure 2:**
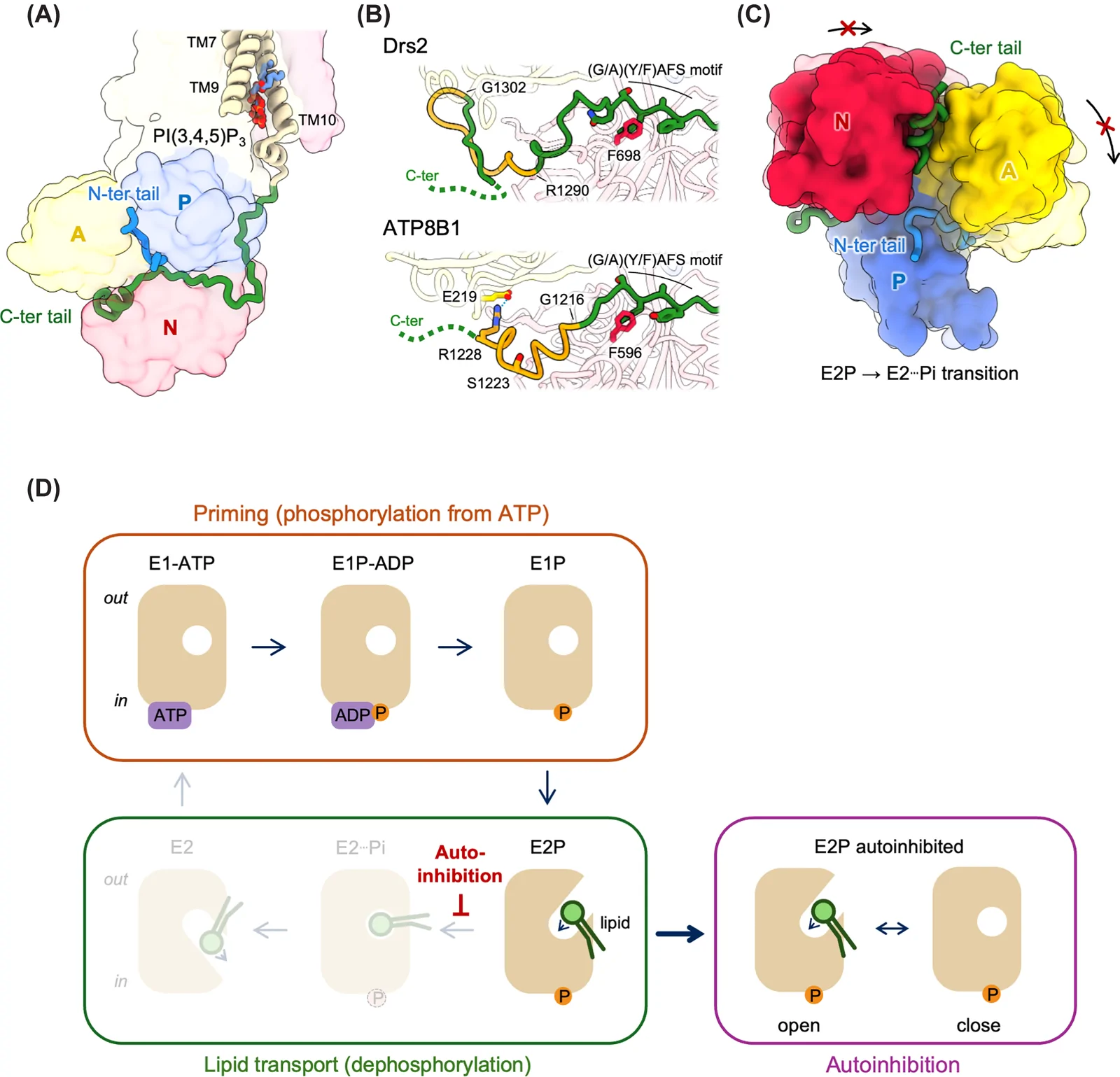
Inhibition of P4-ATPases mediated by their N- and C-terminal tails (**A**) Structure of the human ATP8B1-CDC50A flippase complex trapped in an autoinhibited conformation. ATP8B1 N- and C-terminal tails are bound to the P-, N-, and A-domains, as determined by cryoEM. In this structure, a PI(3,4,5)P_3_ molecule (shown in blue) was identified between TM7, TM9, and TM10. The N- and C-terminal tails of ATP8B1 are colored light blue and green, respectively. Color code as in Figure 1A (PDB: 8OX7 [14]). (**B**) Close-up views of the C-terminal tails interacting with the N- and A-domains of ATP8B1 and Drs2. Both tails adopt a similar organization, with the highly conserved (G/A)(Y/F)AFS motif engaging the ATP-binding pocket of the N-domain. The highly conserved phenylalanine of the N-domain, involved in the adenine base coordination, is shown in red sticks (F698 and F596 for Drs2 and ATP8B1, respectively). The key autoinhibitory regions identified in Drs2 (1290–1302) by Azouaoui et al. [26] and in ATP8B1 (1216–1228) by Laursen et al. [32] are highlighted in orange and mediate the interaction between the A- and N-domains. The E219-R1228 salt bridge in ATP8B1 is shown in blue, with the residues involved shown as sticks. ATP8B1 residue S1223, which may be phosphorylated to relieve autoinhibition, is shown as sticks. The remaining C-terminal part of ATP8B1 and Drs2 tails, unresolved in cryoEM structures, is shown as dashed lines. (PDB: 8OX7 [14] and 6ROH [9]). (**C**) The N- and C-terminal tails prevent A-domain rotation. In the autoinhibited conformation, the C-terminal tail, anchored to the N-domain, prevents the rotation of the A-domain that normally results from the allosteric signal transmitted through TM1 and TM2 upon lipid occlusion. In ATP8B1, sequestration of the A-domain is further reinforced by steric hindrance from the N-terminal tail. The E2P conformation is shown as a surface representation, whereas the E2^…^Pi conformation is shown as a transparent surface. Color code as in Figure 1A (PDB: 8OX7 and 8OXC [14]). (**D**) Autoinhibited P4-ATPase catalytic cycle. After the priming phase, the high-energy E1P conformation transitions into the E2P state, which can be further stabilized by N- and/or C-terminal tails binding. This stabilization prevents the flippase from occluding the lipid substrate. Instead, the protein remains in equilibrium between an outward-open conformation competent for lipid binding and a closed conformation in which the lipid-binding site is inaccessible.

Although Drs2 and ATP8B1 share similar features of autoinhibition, it remains unclear whether this mechanism is conserved across other P4-ATPase lipid flippases. Among the 14 mammalian P4-ATPases, members of the ATP8B (ATP8B1–B4) and ATP8A (ATP8A1–2) subfamilies contain the (G/A)(Y/F)AFS motif in their sequence, suggesting that autoregulation may extend beyond ATP8B1. Consistent with this hypothesis, the activity of ATP8A2 has also been shown to be regulated by its C-terminal tail, although the underlying mechanism appears to differ. Truncation of the entire C-terminal tail increases the maximal ATPase activity of ATP8A2, yet the full-length enzyme remains catalytically active [28]. Moreover, partial truncation of the C-terminal tail, including the (G/A)(Y/F)AFS motif, decreases ATPase activity [34], indicating that this region exerts both positive and negative regulatory effects. In ATP8A1, the C-terminal tail may also play a regulatory role. The tail is ordered only in the E2P state, where it extends through the catalytic domains. Because the full-length protein is catalytically active, it has been proposed that the C-terminal tail maintains the N-domain away from the A-domain in the E2P state, thereby facilitating the rotational rearrangement of the A-domain required for PS binding [13].

Other P4-ATPases, which do not contain the (G/A)(Y/F)AFS motif, do not appear to be regulated in this manner. For example, purified full-length ATP11A, ATP11B, and ATP11C display ATPase activity as well as lipid transport activity when reconstituted in proteoliposomes [35]. Nevertheless, it should be noted that in many cases, ATPase and/or lipid transport activity has not been assessed using truncated variants. Hence, the existence of autoinhibitory mechanisms cannot be completely ruled out.

Interestingly, the presence of polybasic clusters within the N- or C-terminal tails of a subset of P4-ATPases, including ATP8B4, ATP9A, and ATP9B, may render these proteins susceptible to regulation through an electrostatic switch mechanism. In this model, reversible association of the polybasic terminal tails with the negatively charged cytosolic leaflet of the membrane would modulate P4-ATPase activity by controlling the membrane engagement of the autoinhibitory tails [36].

In summary, *in vitro* studies using N- and/or C-terminally truncated variants have provided valuable insights into the extent and molecular basis of P4-ATPase autoinhibition by enabling direct comparison of ATPase and lipid transport activities with those of full-length proteins. Structural studies capturing P4-ATPases at different stages of the catalytic cycle have further contributed to our understanding of how the terminal tails influence P4-ATPase conformation and activity. Nevertheless, despite growing evidence that terminal-tail-mediated autoinhibition is a common regulatory feature of P4-ATPases, a unified molecular mechanism underlying this mode of autoregulation remains to be established.

## Activation by phosphoinositides

Several P4-ATPases are strongly stimulated by PIPs in the presence of their transported lipid substrate. Prominent examples include Drs2 and ATP8B1, whose activities are dependent on phosphatidylinositol 4-phosphate (PI(4)P) and phosphatidylinositol 3,4,5-triphosphate (PI(3,4,5)P_3_), respectively [14,25–27,33,37]. Whereas Drs2 appears to be specific for PI(4)P [25,26], ATP8B1 can be stimulated by all phosphoinositide (PIP) species, including mono-, bis-, and trisphosphorylated PIPs [27]. Nevertheless, ATP8B1 displays a substantially higher apparent affinity for PI(3,4,5)P_3_ than for other PIPs such as PI(4)P or phosphatidylinositol 4,5-bisphosphate (PI(4,5)P_2_). Likewise, the *Plasmodium chabaudi* flippase PcATP2 exhibits enhanced ATPase activity in the presence of PI(4)P [38].

CryoEM structures and molecular dynamics simulations have shown that PIP binding to Drs2 and ATP8B1 occurs within a positively charged cavity formed by TM helices TM7, TM8, and TM10, near the cytosolic face of the membrane (Figure 2A) [9,14]. Interestingly, this binding site closely resembles that identified for phosphatidylinositol 3,5-bisphosphate (PI(3,5)P_2_) in ATP13A2, a member of the P5 subfamily of P-type ATPases that transports polyamines across the lysosomal membrane [39]. A comparable positively charged cavity has also been predicted in plant flippases based on structural modeling [40]. However, whether plant flippases are similarly modulated by PIPs remains to be established experimentally.

Conflicting results have been reported regarding the mechanism by which PIPs activate P4-ATPases, and how PIP binding is coupled to increased lipid-flipping activity remains largely unresolved. Initial studies showed that PI(4)P can directly stimulate both ATP hydrolysis and lipid transport by full-length Drs2 [25,33]. Consistent with this finding, Bai et al. observed that, upon incubation of full-length Drs2 with PI(4)P, the C-terminal tail was no longer visible in the electron density map, suggesting that it had dissociated from the catalytic domains and that PI(4)P binding might be sufficient to relieve autoinhibition [33]. Surprisingly, however, PI(4)P itself was not resolved in the electron density map. Moreover, the activated conformation was obtained only after a prolonged 15-h incubation of the purified protein with PI(4)P at 4°C, raising the possibility that partial proteolysis of the intrinsically disordered C-terminal tail may have occurred during this incubation.

In contrast, several studies reported that, for both ATP8B1 and Drs2, PIPs stimulate ATPase activity *in vitro* only after removal of the autoinhibitory C-terminal tail [26,27,29,30]. Furthermore, cryoEM structures of Drs2 reported by Timcenko et al. revealed that PI(4)P binding promotes the formation of an amphipathic helix immediately downstream of TM10, which generates a pulling force that destabilizes a short α-helical segment of the C-terminal tail [9]. This region overlaps with the previously identified Gea2-binding site, which is known to activate Drs2 [37,41]. Based on these observations, it has been proposed that, *in vivo*, PI(4)P may stimulate Drs2 by partially displacing the C-terminal tail and thereby facilitating its interaction with positive regulators such as Gea2 [9]. These findings further suggest that the stimulatory effect of PI(4)P may be mediated, at least in part, independently of the Drs2 C-terminal tail. PIP binding could, for instance, promote conformational changes within the TMD that enhance lipid transport by facilitating substrate entry into the transport pathway or by stabilizing transport-competent conformations.

An alternative mechanism for ATP8B1 activation was reported by Cheng et al. In this study, bile acids were shown to stimulate the ATPase activity of full-length ATP8B1-CDC50A/B complexes. This activation was abolished upon mutation of a short, positively charged stretch located within an extension of the P-domain that is specific to ATP8B1 orthologs, suggesting that bile acids may bind to this region and thereby destabilize the autoinhibitory N- and C-terminal extensions [31]. The stimulation of ATP8B1 by bile acids is consistent with its localization to the canalicular membrane of hepatocytes, where bile acids synthesized in hepatocytes are secreted into the bile canaliculus. Interestingly, however, bile acids stimulated ATP8B1 activity in the absence of PIPs, suggesting that this regulatory mechanism is distinct from PIP-dependent activation. Further studies will be required to elucidate the molecular mechanism underlying bile acid-mediated activation of ATP8B1 and to determine how this regulatory pathway is integrated with other mechanisms of ATP8B1 activation.

## Activation by interacting proteins

It is noteworthy that autoinhibition by N- and/or C-terminal extensions is a common feature among members of the large P-type ATPase family (e.g., [42–44].). Activation and regulation can depend on proteins interacting with these autoinhibitory regions (Figure 3). For example, plasma membrane Ca^2+^-ATPases (PMCAs) from the P2B-ATPase family are activated by calmodulin binding to a C-terminal calmodulin-binding domain in the presence of elevated Ca^2+^ levels in the cytoplasm. This activation results in PMCA-catalyzed Ca^2+^ efflux and contributes to the maintenance of normal intracellular Ca^2+^ concentrations [45,46]. Plasma membrane H^+^-ATPases from the P3A-ATPase family are activated by the binding of 14-3-3 proteins to the C-terminal tail, an interaction that additionally requires C-terminal phosphorylation [47,48].

**Figure 3:**
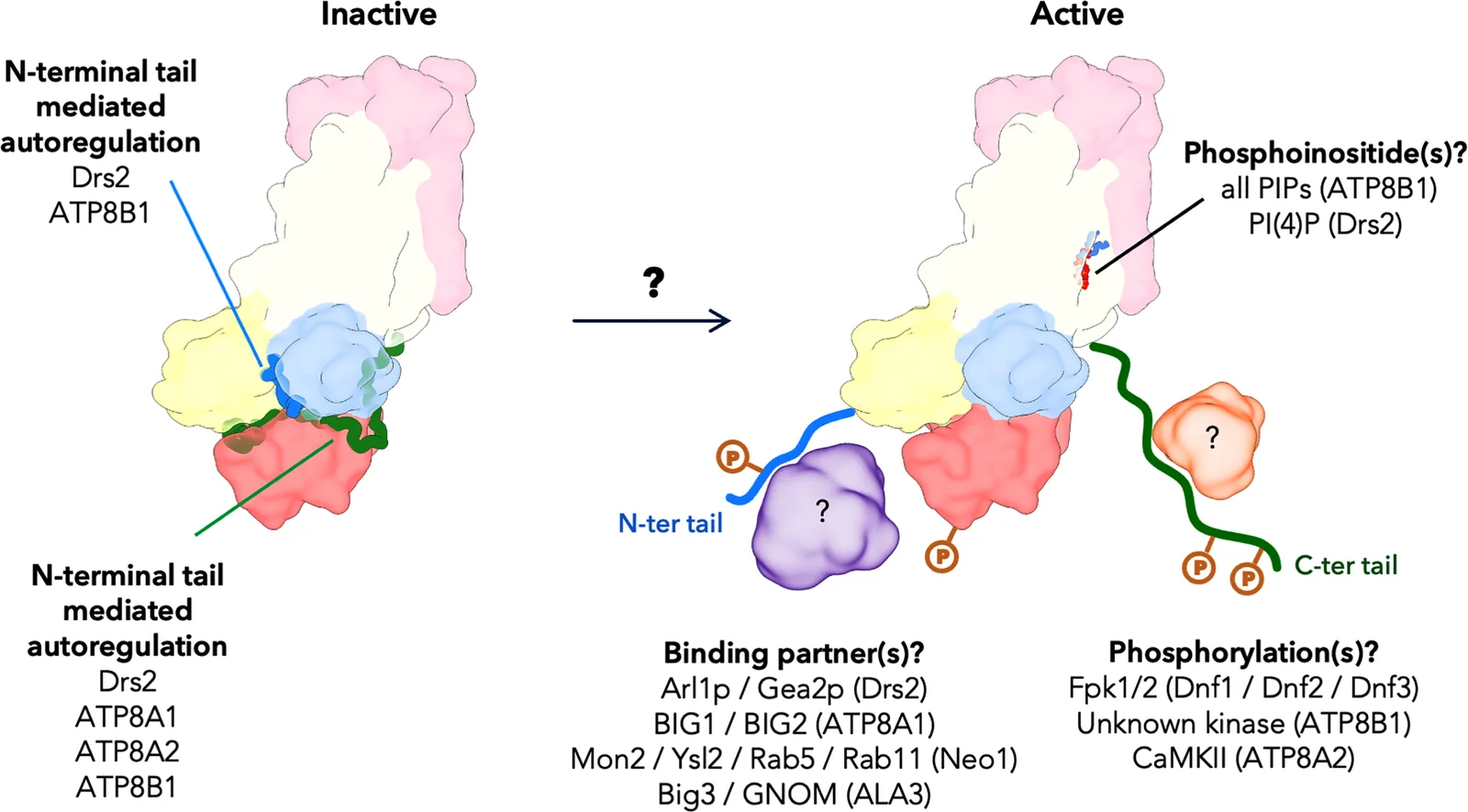
Putative activation mechanisms in P4-ATPases P4-ATPase activity can be regulated through multiple mechanisms involving lipids, proteins, and regulatory phosphorylations. PIP binding, interactions with regulatory protein partners, and phosphorylation have been proposed to modulate flippase activity, at least in part, by promoting displacement of the N- or C-terminal tail and relieving autoinhibition.

Are interactions with partner proteins also a possible activation mechanism of autoinhibited P4-ATPases? Drs2 was found to interact with the small ADP-ribosylation factor (Arf) like (Arl) GTPase Arl1 as well as the Arf guanine nucleotide exchange factor (Arf-GEF) Gea2 [41,49]. Small Arf and Arl GTPases are monomeric G proteins and key regulators of intracellular trafficking [50]. They function as molecular switches, cycling between an inactive GDP-bound state and an active GTP-bound state that binds to effector proteins and initiates downstream signaling pathways. Arf-GEFs catalyze the GDP to GTP exchange on small Arf GTPases through their conserved catalytic Sec 7 domain [51,52].

Interestingly, Arl1 binds to the N-terminus of Drs2, while Gea2 interacts with a stretch in the C-terminal tail through its catalytic Sec 7 domain, suggesting that both proteins, by binding to the terminal extensions of Drs2, are involved in autoinhibition release and activation [41,49]. Using a fluorescence-based lipid transport assay in isolated TGN membranes, it was shown that both the interaction of Gea2 and Arl1 with Drs2, as well as the interaction between Gea2 and Arl1, are indispensable for Drs2 flippase activity [37,49]. This supports the notion of a tripartite complex whose formation is required for Drs2 activation and/or regulation of Drs2-mediated lipid transport. Consistent with this model, the structure of Gea2 in complex with Arl1 has been resolved by cryoEM [53], providing mechanistic insights into how such a complex could form. The case of Drs2, Arl1, and Gea2 exemplifies a possible activation mechanism in which a small GTPase and a GEF in concert modulate flippase activity.

Direct interactions between a flippase and an Arf-GEF were recently also found for ATP8A1 [34,54]. The C-terminal tail of ATP8A1 interacts with the catalytic Sec 7 domain of the mammalian Arf-GEFs BIG1 and BIG2. This interaction enhances the catalytic GEF activity of BIG1 and BIG2 at the TGN, resulting in an enhanced activation of small Arf GTPases [54]. However, a direct interaction of ATP8A1 with small Arf GTPases, as in the case of Drs2 and Arl1, has not been investigated in this study. Similarly, it remains unclear whether BIG1 and BIG2, possibly in combination with small Arf GTPases, are required for ATP8A1-mediated lipid transport activity.

Notably, several other P4-ATPases that are not subject to autoinhibition by their terminal tails were reported to interact with small GTPases and their cognate GEFs. The yeast flippase Neo1 physically and genetically interacts with the Arf-GEF Mon2/Ysl2 and also shows a genetic interaction with the small GTPase Arl1 [55]. Mon2/Ysl2 has likewise been shown to physically interact with Arl1 [56], raising the possibility that these proteins form a tripartite complex analogous to the proposed Drs2-Gea2-Arl1 complex. Yet, the impact of these interactions on Neo1 flippase activity remains to be determined. The plant flippase ALA3 physically and genetically interacts with the Arf-GEFs BIG3 and GNOM and has been proposed to regulate their activity [57]. Conversely, whether these Arf-GEFs reciprocally regulate ALA3 activity has not yet been investigated. Mammalian ATP9A associates with Mon2/Ysl2 and binds the Rab GTPases Rab5 and Rab11, potentially contributing to the regulation of Rab GTPase activation [58,59]. However, whether these interactions modulate ATP9A flippase activity remains unknown.

Although in the aforementioned cases the exact interaction sites were not determined, nor whether and how these interactions affect flippase-catalyzed lipid transport, these examples suggest that the interplay between flippases, small GTPases, and GEFs is evolutionarily conserved. To further elucidate the molecular mechanisms by which GEFs and small GTPases regulate or activate flippase-mediated lipid transport, *in vitro* interaction studies using purified components could provide valuable insight into whether these interactions are direct, their binding affinities, or whether regulation occurs through the formation of a stable complex or a sequence of transient binding events. In addition, structural studies, molecular modeling, or molecular dynamics simulations may help to identify the precise interaction interfaces involved.

## Activation by phosphorylation

In addition to activation by PIPs and interacting proteins, P4-ATPase flippases can also be regulated by post-translational modifications, and more specifically by phosphorylation. This mechanism was first described in the yeast flippases Dnf1 and Dnf2, when Nakano et al. [60] identified two serine/threonine protein kinases, Fpk1 and Fpk2, as upstream activators of these flippases. In the double *fpk1Δfpk2Δ* mutant, phospholipid uptake was severely impaired, suggesting that Fpk1/Fpk2-dependent phosphorylation is required for Dnf1 and Dnf2 activity. This was further supported by cryoEM data, which revealed that all 17 known phosphorylation sites are located exclusively in disordered regions of Dnf1—the N-terminal tail, an internal loop between T290 and A378, and the C-terminal tail—suggesting that phosphorylation relieves autoinhibition rather than directly modulating the catalytic core [61]. This mechanism was subsequently extended to Dnf3, following the work of Frøsig et al [62].

Adding a further layer of complexity, this regulatory pathway is embedded in a broader upstream kinase cascade. Ypk1, another serine/threonine kinase involved in the response to plasma membrane stress, endocytosis, and cell wall integrity [63,64], phosphorylates and thereby down-regulates Fpk1 activity, placing Dnf1/Dnf2 activation under the control of a multi-tiered signaling network. Furthermore, complex sphingolipids have been shown to counteract Ypk1-mediated inhibition of Fpk1, linking flippase activity to the lipid composition of the membrane itself [65].

A similar mechanism has been described for the human P4-ATPase ATP8B1. Large-scale phosphoproteomics studies identified a phosphorylation site at S1223 in the C-terminal tail of mouse ATP8B1 [66]. Using synthetic peptides mimicking the phosphorylated and non-phosphorylated C-terminal tail, phosphorylation at S1223 was shown to dramatically impair the inhibitory capacity of this region, shifting the IC50 from ∼22 to ∼380 μM. This effect was further amplified in constructs retaining the N-terminal tail, where inhibition was ∼270-fold more efficient (IC50 ∼0.08 μM), underscoring the cooperative nature of the N- and C-terminal autoinhibitory mechanism [27]. Phosphorylation at S1223 similarly reduced the inhibitory effect in this context, demonstrating that a single phosphorylation event is sufficient to significantly relieve autoinhibition.

A further example of phosphorylation-dependent regulation has been described for the mammalian P4-ATPase ATP8A2. Unlike its yeast ortholog Drs2, ATP8A2 is insensitive to PI(4)P-mediated activation [34]. Instead, sequence analysis of its C-terminal domain revealed a conserved calcium/calmodulin-dependent protein kinase II (CaMKII) consensus motif, with S1138 as the phosphorylation target residue. Site-directed mutagenesis and *in vitro* phosphorylation assays confirmed that S1138 is specifically phosphorylated by CaMKII, as the S1138A mutant showed greater than 10-fold reduced phosphorylation compared with wild-type ATP8A2. Functionally, the S1138A substitution reduced PS-stimulated ATPase activity by ∼33%, while the phosphomimetic S1138D mutant retained wild-type-like activity, strongly suggesting that phosphorylation at this residue is required for full flippase activity [34]. This phosphorylation site is located within an autoinhibitory domain of the C-terminal tail, known to inhibit ATP8A2 activity. One plausible model is that CaMKII-mediated phosphorylation at S1138 may disrupt autoinhibition, thereby placing ATP8A2 regulation under indirect calcium control, as CaMKII is activated by elevated intracellular Ca^2+^.

## Concluding remarks

In recent years, advances in structural biology, biochemistry, and cell biology have considerably expanded our understanding of the molecular mechanisms governing P4-ATPase function. It is now clear that a number of P4-ATPases, if not all, are regulated through multiple, interconnected mechanisms, including autoinhibition, PIP binding, protein–protein interactions, and post-translational modifications. A major challenge for the future will be to determine how these regulatory inputs are integrated within the cellular environment to control flippase activity in space and time. Beyond these regulatory mechanisms, another important question concerns the breadth of substrate recognition. Although beyond the scope of the present review, recent studies indicate that the lipid transport specificity of P4-ATPases may be considerably broader than previously anticipated [14,30,67,68]. Carefully reassessing substrate specificity using purified, reconstituted systems will therefore be essential to establish the physiological repertoire of these lipid flippases. Such efforts will not only advance our understanding of membrane lipid asymmetry but may also facilitate the development of therapeutic strategies targeting P4-ATPase dysfunction in human disease.

## Perspectives

- The exquisite spatial organization of lipids within and between cellular membranes is a fundamental determinant of membrane identity, signaling, and cellular function. Accordingly, defects in the proteins that establish and maintain transbilayer lipid asymmetry, including P4-ATPase lipid flippases, are associated with a broad spectrum of human diseases.
- Recent cryoEM structures of P4-ATPases, together with *in silico*, biochemical, and cellular studies, have defined a common structural framework while uncovering an unexpected diversity of regulatory mechanisms.
- A major challenge for the field will be to define the mechanistic basis of P4-ATPase activation by PIPs, protein binding partners, and regulatory phosphorylations. Addressing this question will require an integrated approach combining cryoEM, molecular dynamics simulations, biochemical reconstitution, and cellular studies.

## Open Access

Open access for this article is provided via our Subscribe to Open programme, made possible through the support of our subscribing institutions. Learn more here: https://portlandpress.com/pages/open_access_options_and_prices#conditional.

## Abbreviations

A: actuator
Arf: ADP-ribosylation factor
Arl: Arf-like
Arf-GEF: Arf guanine nucleotide exchange factor
CaMKII: calcium/calmodulin-dependent protein kinase II
cryoEM: cryo-electron microscopy
GEF: guanine nucleotide exchange factor
N: nucleotide binding
P: phosphorylation
PI: phosphatidylinositol
PI(3,4,5)P_3_: phosphatidylinositol 3,4,5-trisphosphate
PI(3,5)P_2_: phosphatidylinositol 3,5-bisphosphate
PI(4)P: phosphatidylinositol 4-phosphate
PI(4,5)P_2_: phosphatidylinositol 4,5-bisphosphate
PIPs: phosphoinositides
PMCA: plasma membrane Ca^2+^-ATPase
PS: phosphatidylserine
TM: transmembrane
TMD: transmembrane domain
